# microRNA cluster MC‐let‐7a‐1~let‐7d promotes autophagy and apoptosis of glioma cells by down‐regulating STAT3

**DOI:** 10.1111/cns.13273

**Published:** 2019-12-23

**Authors:** Zhuan‐Yi Yang, Ying Wang, Qing Liu, Ming Wu

**Affiliations:** ^1^ Department of Neurosurgery Xiangya Hospital Central South University Changsha China; ^2^ Department of Pathology Xiangya Medical School of Central South University & Xiangya Hospital Central South University Changsha China

**Keywords:** apoptosis, autophagy, glioma, microRNA cluster MC‐let‐7a‐1 ~ let‐7d, STAT3

## Abstract

**Background:**

Accumulating evidence has highlighted the correlation between microRNAs (miRNAs) and the progression of glioma. However, the role of miR cluster MC‐let‐7a‐1 ~ let‐7d in glioma remains elusive. Thus, the current study aimed to investigate the effect of miR cluster MC‐let‐7a‐1 ~ let‐7d on glioma progression.

**Methods and Results:**

Microarray data analysis provided data indicating the involvement of miR cluster MC‐let‐7a‐1 ~ let‐7d in glioma via STAT3. The expression of let‐7a‐1, let‐7d, let‐7f‐1, and miR cluster MC‐let‐7a‐1 ~ let‐7d was diminished in the glioma tissues and the cell lines. Additionally, our results revealed that STAT3 was a target gene of let‐7d, let‐7a‐1, and let‐7f‐1, which was further verified by the dual‐luciferase reporter gene assay. Moreover, STAT3 expression was negatively mediated by let‐7a‐1, let‐7d, and let‐7f‐1. Up‐regulated miR cluster MC‐let‐7a‐1 ~ let‐7d or silenced STAT3 suppressed cell proliferation but accelerated cell apoptosis and autophagy. Moreover, restrained tumor growth was identified in the nude mice treated with miR cluster MC‐let‐7a‐1 ~ let‐7d mimics or STAT3 siRNA.

**Conclusion:**

Taken together, the miR cluster MC‐let‐7a‐1 ~ let‐7d promotes glioma cell autophagy and apoptosis by repressing STAT3. The current study highlights the potential of the miR cluster MC‐let‐7a‐1 ~ let‐7d as biomarkers and promising treatment strategies for glioma.

## 1 INTRODUCTION

As one of the most common intracranial tumors, glioma can occur anywhere across the central nervous system (CNS), with a large portion of cases afflicting the brain and glial tissues. Of note, glioblastoma (GBM) accounts for approximately 45% of all cases of glioma.^1^ Glioma is often accompanied by poor patient prognosis particularly in those diagnosed with high‐grade glioma.^2^ From a histological perspective, gliomas share similarities with glial cells such as oligodendrocytes and astrocytes.^3^ The current treatment approaches for glioma are generally comprised of maximal safe resection, followed by concomitant temozolomide with (TMZ) or external beam radiation combined with maintenance chemotherapy, with newly diagnosed patients generally having a 12‐ to 18‐month median overall survival period.^4^ On the one hand, as a cellular survival mechanism, autophagy is associated with the catabolic degradation of damaged organelles and proteins during stress, while on the other hand, it has been shown to accelerate tumor progression through regulating cell survival.^5^ For instance, it has been demonstrated that activation of autophagy suppresses the resistance to radiation of glioma‐initiating cells (GICs), which is often employed in anti‐cancer therapy of GBM.^6^ However, to our knowledge, malignant gliomas are widely considered to be highly recurrent tumors even after surgery, chemotherapy, radiation, and immunotherapy approaches. In the last decades, the treatment strategies for gliomas have not changed appreciably because of the limited understanding of the biology of the disease.^7^ Thus, it is imperative to deepen our knowledge regarding the finer details of the molecular mechanisms of autophagy in the progression of glioma, in order to identify more effective therapies.

Signal transducers and activators of transcription 3 (STAT3) have been closely correlated with carcinogenesis via intervention of the transcription of critical genes which control cell differentiation, apoptosis, proliferation, and angiogenesis.^8^ Zhang et al revealed that STAT3 is expressed at a high level in glioma cells, while further indicating that the down‐regulation of STAT3 suppresses tumor growth of mice with glioma.^9^ As short noncoding endogenous RNAs, microRNAs (miRNAs) have been validated to engage in the glioma progression.^10^ For example, ectopic expression of let‐7d is involved in the suppressive role of Ficus carica Latex in GBM cell invasion.^11^ Furthermore, a study has indicated the suppressive influence of let‐7a over‐expression on glioma cell malignancy.^12^ Additionally, let‐7f targets periostin has been reported to repress glioma cell invasion, migration, and proliferation.^13^ The human miR cluster MC‐let‐7a‐1 ~ let‐7d, including let‐7d, let‐7f‐1, and let‐7a‐1, has been shown to exhibit low levels in hepatocellular carcinoma (HCC) cells.^14^ Bioinformatics website (http://www.mirdb.org/) presented data indicating that there were putative binding sites between let‐7d, let‐7a‐1, and let‐7f‐1 and 3’‐untranslated region (3’UTR) of STAT3. Moreover, STAT3 has been reported to be a target of let‐7a in a previous study.^15^ Based on the aforementioned exploration of literature, we subsequently proposed a hypothesis that miR cluster MC‐let‐7a‐1 ~ let‐7d may be involved in glioma development by regulating STAT3. Hence, the study was designed to investigate the mechanism by which the miR cluster MC‐let‐7a‐1 ~ let‐7d influences the autophagy of glioma cells in connection to STAT3.

## 2 MATERIALS AND METHODS

### 2.1 Ethics statement

The study protocol was permitted by the Ethics Committee of Xiangya Hospital Central South University. Informed written consent was attained from all participants (Approval number: 201211002). Animal use and experimental steps were conducted in line with animal ethical standard. Extensive efforts were made in order to minimize animal usage and suffering (Approval number: 201303004).

### 2.2 Bioinformatics analysis

The microarray gene datasets related to glioma were downloaded from the National Center for Biotechnology Information (NCBI). The microarray data of the dataset http://www.ncbi.nlm.nih.gov/geo/query/acc.cgi?acc=GSE12657 retrieved from Gene Expression Omnibus (GEO) were downloaded including five cases of normal samples and seven cases of glioma samples. On the basis of limma package of Bioconductor, R language combined with the empirical Bayes method was utilized to sort out the differentially expressed genes (DEGs). Finally, the DEGs were identified using the limma package based on the cutoff of |log2 (fold change)|>2 and adjusted *P* value < .05. The miRNAs targeting STAT3 were predicted using the tools of microRNAmap (http://mirnamap.mbc.nctu.edu.tw/microRNAmap), TargetScan (http://www.targetscan.org/vert_71/TargetScan), and mirDIP (http://ophid.utoronto.ca/mirDIP/mirDIP). The miR cluster was retrieved from the miRBase dataset (http://www.mirbase.org/cgi-bin/mirna_entry.pl?acc=MI0000098).

### 2.3 Study subjects

One‐hundred thirty‐two cases of glioma tissues were collected from patients diagnosed with gliomas who had undergone surgery at the general surgical department of Xiangya Hospital Central South University from December 2012 to October 2017. The detailed clinical information is listed in Table S1. The tissues with hemorrhage, necrosis, and electrocautery were immediately excluded from the study. All cases were confirmed as glioma based on pathological examination. No patient received any treatment of glioma in the past 3 months and had complete clinical data. In addition, 20 brain samples were obtained from patients receiving intracranial decompression treatment for hypertensive cerebral hemorrhage during the same period at Xiangya Hospital Central South University. Among the normal controls, 12 were male and eight were female with a median age of 41 years, ranging from 17 to 65 years. The adjacent normal brain tissues from the nonfunctional areas were collected during the operation. All samples were frozen in cryopreservation tubes and stored in a −80°C refrigerator for further use. Quantitative detection of miRNA and STAT3 in tissues was conducted by reverse transcription‐quantitative polymerase chain reaction (RT‐qPCR).

### 2.4 Screen of cell line

Human normal glial cell line HEB, human glioma cell lines (U87 and U251), and human glioma cell line SHG44 were purchased from Yan‐Yu Biotechnology Co., Ltd., Cell Bank, Shanghai Institutes for Biological Sciences, Chinese Academy of Sciences (Shanghai, China), and American Type Culture Collection (ATCC), respectively. All cell lines were cultured in Dulbecco's Modified Eagle Medium (DMEM, Gibco) containing 10% fetal bovine serum (FBS, Hangzhou Sijiqing Biological Engineering Materials Co., Ltd.), 100 mg/L streptomycin and 100 U/L penicillin (Gibco) at 37°C with 5% CO_2_. The cells were passaged once every 2‐3 days, with the cells at the logarithmic growth phase selected for subsequent experimentation. The expression of let‐7d, let‐7a‐1 and let‐7f‐1 in glioma cell lines was determined by RNA isolation and quantification.

### 2.5 Cell transfection and culture

The cells were treated with 0.25% trypsin and then passaged at a ratio of 1:2 or 1:3. The cells at passage of 3‐4 in the logarithmic growth phase were resuspended in DMEM to adjust the cell density into 1 × 10^6^ cells/mL. The cells were then plated into 6‐well plates. After 24 hours, NC mimic, let‐7a‐1 mimic, let‐7d mimic, let‐7f‐1 mimic, cluster mimic, NC inhibitor, let‐7a‐1 inhibitor, let‐7d inhibitor, let‐7f‐1 inhibitor, or cluster inhibitor was delivered into the cells in accordance with the instructions of lipofectamine 2000 (Invitrogen). The medium was changed 6 hours after transfection, and the cells were collected 48 hours later for subsequent experiments.

### 2.6 5‐Ethynyl‐2’‐deoxyuridine (EdU) assay

The cells at the logarithmic growth phase were incubated in the 24‐well plate with 250 μL culture medium and 10 μL working solution for 45 minutes. After removal of the solution, the cells were washed three times with phosphate‐buffered saline (PBS) and fixed in 3.7% formaldehyde/PBS at room temperature for 15 minutes. After the fixation solution had been removed, the cells were rinsed twice with 3% bovine serum albumin (BSA)/PBS and permeated with 250 μL PBS supplemented with 0.5% Triton X‐100 at room temperature for 20 minutes. Next, 10 × storage solution was diluted into 1 × Click‐iT EdU with ddH_2_O to prepare Click‐iT reaction mixture, which was utilized within 15 minutes after preparation. For each well, 250 µL Click‐iT reaction mixture was supplemented for a 30‐minute incubation at room temperature under conditions void of light. Following discarding of the Click‐iT reaction mixture, the cells were rinsed with 3% BSA/PBS, stained with 4',6‐diamidino‐2‐phenylindole (DAPI), and mounted. The EdU staining cells were analyzed and counted under the fluorescent microscope.

### 2.7 Flow cytometry

The cells were detached with trypsin and collected into a 15‐mL centrifuge tube. The cells were centrifuged at 800 *g*, after which the precipitation was washed twice with PBS. The cells were subsequently resuspended with 500 μL binding buffer based on the protocols of the Annexin V‐fluorescein isothiocyanate (FITC) Apoptosis Detection Kit I (556547, BD Biosciences). The cells were then permitted to react with 5 µL Annexin V‐FITC and 5 µL propidium iodide (PI) for 15 minutes under dark conditions. Finally, a flow cytometer was employed to detect cell apoptosis.

### 2.8 Immunofluorescence staining

For the LC3‐green fluorescent protein (GFP) experiments, U87 cells were incubated with DMEM plus 10% FBS in 6‐well plates at 37℃ with 5% CO_2_. When the cells were covered with 80% microscopic view, they were subsequently incubated in Opti‐minimal essential medium (MEM; Thermo Fisher Scientific Inc) and transfected with GFP‐LC3 vector (GeneChem Co., Ltd.) with Lipofectamine 2000 (Invitrogen Inc). Afterward, the cells were fixed with 4% paraformaldehyde and mounted using Vectashield with DAPI. The images were recorded under a fluorescent microscope (Nikon TE2000; Nikon Instruments Inc), with the number of GFP‐LC3 punctate dots in the GFP‐LC3–positive cells determined (five punctate dots).^16^

### 2.9 Dual‐luciferase reporter gene assay

The target genes for let‐7a‐1, let‐7d, and let‐7f‐1 were predicted at https://cm.jefferson.edu/rna22/Interactive/, and whether STAT3 was a target gene of let‐7a‐1, let‐7d, and let‐7f‐1 was further validated by dual‐luciferase reporter gene assay. The 3’UTR fragments of STAT3 were artificially synthesized and inserted into pGL3‐control (Promega), that was pGL3‐STAT3‐wild type (WT). The mutations of the 3’UTR were generated, after which the mutant sequence was inserted into the pGL3‐control, referred to as pGL3‐STAT3–mutant type (MUT). The two aforementioned plasmids were transfected with let‐7a‐1 mimic, let‐7d mimic, or let‐7f‐1 mimic into HEK‐293T cells, respectively (Shanghai Institutes for Biological Sciences, CAS, Shanghai, China). Meanwhile, the Renilla luciferase expressing vector pRL‐TK was cotransfected as the internal control for the detection of luciferase activity. The cells were then lysed after 48 hours. The luciferase activity was subsequently detected in the LuminometerTD‐20/20 detector (E5311, Promega) using the Dual‐Luciferase Reporter Assay System Kit (Promega).

### 2.10 RNA isolation and quantitation

The total RNA of the glioma cells was extracted in strict accordance with the directions of the RNA extraction kit (Invitrogen). The primers of let‐7d, let‐7a‐1, STAT3, let‐7f‐1, U6, and β‐actin were synthetized by TaKaRa Biotechnology Co., Ltd. The cDNA was PCR amplified using the PrimeScript reverse transcription kit with primers specific for let‐7a‐1, 5′‐TTTCTATCAGACCGCCTGGATGCAGACTTT‐3′ (forward) and 5′‐GATTCCTTTTCACCATTCACCCTGGATGTT‐3′ (reverse); let‐7d, 5′‐TGAGGTAGTTGGTGGTATGGTT‐3′ (forward) and 5’‐GCGAGCACAGAATTAATACGAC‐3′ (reverse); let‐7f‐1, 5′‐CCGCTCGAGACCCAGCCATGTTCAGTTCT‐3′ (forward) and 5′‐CCCAAGCTTCAGTGAAGAGAACACCAGG‐3′ (reverse); U6, 5′‐GCTTCGGCAGCACATATACTAAAAT‐3′ (forward) and 5′‐CGCTTCACGAATTTGCGTGTCAT‐3′ (reverse). The fluorescence quantitative polymerase chain reaction (PCR) was performed in accordance with the SYBR^®^ Premix Ex Taq^™^ II kit instructions in ABI PRISM^®^ 7300 system. STAT3 primers were 5′‐CAGCCTCTCTGCAGAATTCAA‐3′ (forward) and CD44‐R 5′‐AGCCCATGTGATCTGACACC‐3′ (reverse). β‐actin primers were 5′‐CTGGCACCACACCTTCTACAAT‐3′ (forward) and 5′‐AATGTCACGCACGATTTCCCGC‐3′ (reverse). U6 was regarded as the internal reference of let‐7a‐1, let‐7d, and let‐7f‐1, while β‐actin was used as the internal reference of STAT3, with the relative expression of the target genes measured using the 2^−ΔΔCt^ method.

### 2.11 Western blot analysis

Total protein was extracted from the cells with the protein concentration assessed using a bicinchoninic acid (BCA) kit. The protein was added to 5 × sodium dodecyl sulfate (SDS) loading buffer and denatured at 95°C for 5 minutes. The protein was isolated with SDS‐polyacrylamide gel electrophoresis (PAGE) and transferred onto the membrane. The membrane was blocked with 5% skimmed milk powder at 4°C overnight. The membrane was rinsed with tris‐buffered saline/Tween 20 (TBST), and incubated with mouse monoclonal antibody to c‐Caspase‐3 (ab2302, 1:1000), t‐Caspase‐3 (ab13585, 1:1000) and P62 (ab56416, 1:1000), and rabbit monoclonal antibody to B‐cell lymphoma‐2 (Bcl‐2; ab32124, 1:1000), LC3B (ab51520, 1:1000), and β‐actin (ab8224, 1:1000) at 4°C overnight. The membrane was then rinsed with TBST, followed by incubation with the secondary antibody labeled with horseradish peroxidase (HRP) at 37°C for 1 hour. All the above antibodies were purchased from Abcam Inc. The blots were developed using enhanced chemiluminescence (ECL) reagents and analyzed using the ImageJ.

### 2.12 In vivo tumorigenicity

Male BALB/c nude mice (n = 112, aged 5 weeks, weighing 18‐20 g) were obtained from the Shanghai laboratory animal center (Shanghai, China). The glioma cells at the logarithmic growth phase were treated with NC mimic, let‐7a‐1 mimic, let‐7d mimic, let‐7f‐1 mimic, cluster mimic, NC inhibitor, let‐7a‐1 inhibitor, let‐7d inhibitor, let‐7f‐1 inhibitor, cluster inhibitor, shRNA‐NC, sh‐STAT3, NC inhibitor + shRNA‐NC, and cluster inhibitor + sh‐STAT3, respectively. The stably transfected U87 cells were then resuspended with PBS into a cell suspension at 2 × 10^7^ cells/mL. The mice were subcutaneously injected with 100 μL of the aforementioned suspension into the proximal axilla of the hind limb, with eight mice placed in each group. The tumor volume (*V*) was measured weekly using vernier calipers and calculated based on the following formula $V\left({\text{mm}}^{3}\right)=\text{width}{\left(W\right)}^{2}\times \text{length}\left(L\right)\times 0.52$.^17^ In the tumorigenic experiment, no nude mice death took place with a tumorigenic rate of 100% (112/112). All mice were euthanized on the 35th day, and their respective tumors were excised with three tumors used for each treatment.

### 2.13 Statistical analysis

All statistical analyses were performed using SPSS 21.0 software (IBM Corp.). Measurement data were expressed as the mean ± standard deviation. The significance of differences was determined using a Student *t* test for single comparison, while data for multiple comparisons were analyzed by one‐way analysis of variance (ANOVA). Data with normal distribution were examined using the Kolmogorov‐Smirnov method. The post hoc test of data with normal distribution was performed by Tukey in multiple comparisons of one‐way ANOVA, and the post hoc test of data with skewed distribution was conducted using Dunn's multiple comparisons in Kruskal‐Wallis test. ANOVA of repeated measurement was used in line charts. All experiments were repeated at least three times. *P* < .05 indicated statistical significance.

## 3 RESULTS

### 3.1 miR cluster MC‐let‐7a‐1 ~ let‐7d is associated with glioma by targeting STAT3

**Figure 1:**
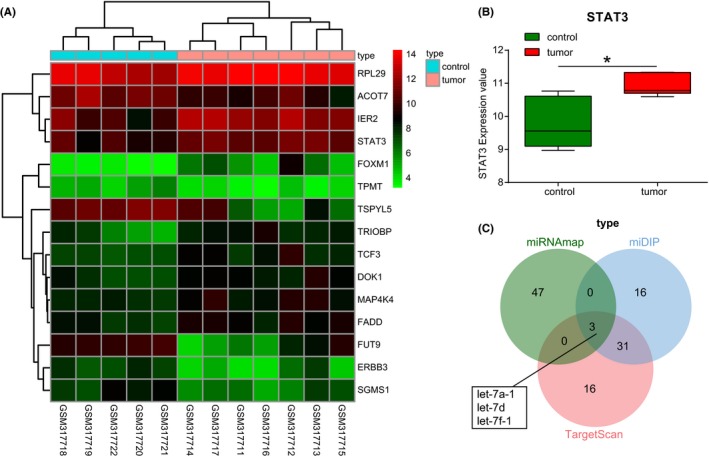
miR cluster MC‐let‐7a‐1 ~ let‐7d targets STAT3 to regulate autophagy and apoptosis of glioma cells. A, the heatmap of DEGs screened from the dataset of http://www.ncbi.nlm.nih.gov/geo/query/acc.cgi?acc=GSE12657. The *x*‐axis represents the sample number, and the *y*‐axis represents the gene name. The right color histogram represents the gene expression, in which the red ones represent the high expression and the green ones represent the low expression. Each rectangle in the figure represents the gene expression in a sample, the left dendrogram represents the gene expression cluster, and the upper dendrogram represents the sample cluster. B, profile analysis of STAT3 gene expression. Green represents STAT3 expression of normal samples and red represents STAT3 expression of glioma samples (adj.*P* < .05). C, Venn analysis of predicted results of miRNAs targeting STAT3. Blue represents the predicted results of mirDIP database, red represents the predicted results of TargetScan database, and green represents the predicted results of miRNAmap database. STAT3, signal transducers and activators of transcription 3; DEGs, differentially expressed genes

The dataset http://www.ncbi.nlm.nih.gov/geo/query/acc.cgi?acc=GSE12657 was downloaded from the GEO database. There were 1941 DEGs that were screened out from the dataset, with 964 of which were up‐regulated genes while 977 of them were down‐regulated genes. The heatmap of DEGs was subsequently plotted (Figure 1A). STAT3 exhibited markedly up‐regulated levels in glioma (adj. *P* = .01900; Figure 1B). Down‐regulated STAT3 has been reported to effectively enhance autophagy as well as the apoptosis of GBM cells in orthotopic transplanted rat samples, while blockade of the STAT3 signaling pathway may also trigger the apoptosis of glioma cells.^18^, ^19^ The upstream regulatory miRNAs of STAT3 were explored by the bioinformatics websites. The top 50 miRNAs targeting STAT3 were selected. Three intersected miRNAs, let‐7d, let‐7a‐1, and let‐7f‐1 were identified (Figure 1C). Evidence was obtained by exploration of the miRBase website indicating that let‐7d, let‐7a‐1, and let‐7f‐1 belonged to the miR cluster MC‐let‐7a‐1 ~ let‐7d, which was selected for the subsequent experimentation. The let‐7 family is generally considered to be a cancer suppressor which has been evidenced by its distinct down‐regulation in a variety of tumors.^11^, ^20^, ^21^, ^22^ Taken together, miR cluster MC‐let‐7a‐1 ~ let‐7d may have the potential to regulate glioma cell autophagy and apoptosis by targeting STAT3 in glioma. In the subsequent experiment, we intended to verify whether the miR cluster MC‐let‐7a‐1 ~ let‐7d participated in autophagy and apoptosis of glioma cells by targeting STAT3.

### 3.2 Screening of representative glioma cell line

**Figure 2:**
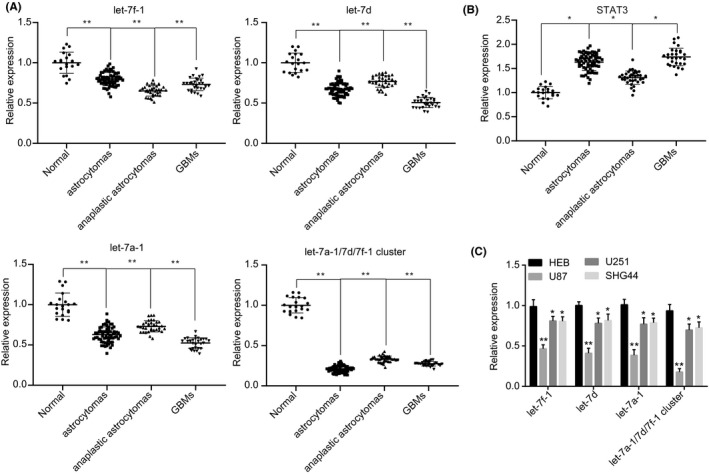
U87 cell line is screened for the following experiments. A, the expression of let‐7a‐1, let‐7d, let‐7f‐1, and miR cluster MC‐let‐7a‐1 ~ let‐7d in three types of glioma (n = 132; astrocytoma, anaplastic astrocytoma, and glioblastoma) and adjacent normal brain tissues (n = 20) randomly collected from December 2012 to October 2017 determined by RT‐qPCR. **P < .01 vs adjacent normal brain tissues. B, the expression of STAT3 in three types of glioma (n = 132, in which n = 68 in astrocytoma, n = 35 in anaplastic astrocytoma, and n = 29 in glioblastoma) and adjacent normal brain tissues (n = 20) determined by RT‐qPCR. *P < .05 or **P < .01 vs adjacent normal brain tissues. C, the expression of let‐7a‐1, let‐7d, let‐7f‐1, and miR cluster MC‐let‐7a‐1 ~ let‐7d in HEB, U87, U251, and SHG44 cells. *P < .05, vs HEB cells. **P < .01. The cell experiment was repeated 3 times. The measurement data were expressed as mean ± standard deviation. Comparison between two groups was analyzed using unpaired *t* test, and comparisons among multiple groups were tested by one‐way analysis of variance (ANOVA)

A total of 132 cases of glioma tissues and 20 cases of nontumor brain tissues were randomly selected from December 2012 to October 2017 in Xiangya Hospital Central South University. According to the tissue source, we divided the samples into three types of glioma: astrocytoma, anaplastic astrocytoma, and glioblastoma and detected the expression of let‐7a‐1, let‐7d, let‐7f‐1, and miR cluster MC‐let‐7a‐1 ~ let‐7d using RT‐qPCR. As depicted in Figure 2A, the expression of let‐7a‐1, let‐7d, and let‐7f‐1 was lower in glioma tissues relative to that of the adjacent normal brain tissues, whereby the miR cluster MC‐let‐7a‐1 ~ let‐7d exhibited the lowest expression pattern (*P* < .01). Concurrently, we detected the expression of STAT3 between the three types of glioma tissues and the adjacent normal tissues by RT‐qPCR. The results showed that STAT3 expression was increased in the three types of glioma tissues (Figure 2B). Compared with HEB cells, the glioma cell lines U87, U251, and SHG44 displayed lower expression of let‐7a‐1, let‐7d, let‐7f‐1, and miR cluster MC‐let‐7a‐1 ~ let‐7d. Among the cell lines, U87 exhibited the lowest expression of miR cluster MC‐let‐7a‐1 ~ let‐7d (*P* < .01; Figure 2C). Hence, based on the results obtained, the U87 cell line was selected for the following experiments.

### 3.3 Over‐expression of miR cluster MC‐let‐7a‐1 ~ let‐7d inhibits cell proliferation in glioma

**Figure 3:**
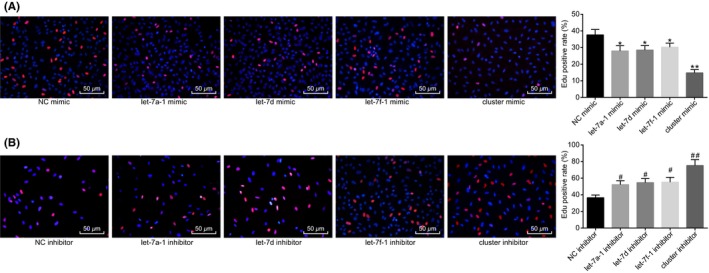
Glioma cell proliferation is repressed by over‐expression of miR cluster MC‐let‐7a‐1 ~ let‐7d. A, cell proliferation after over‐expression of let‐7a‐1, let‐7d, let‐7f‐1, or miR cluster MC‐let‐7a‐1 ~ let‐7d evaluated by the EdU assay (200×). *P < .05, **P < .01 vs U87 cells treated with NC mimic. B, cell proliferation assessment after inhibition of let‐7a‐1, let‐7d, let‐7f‐1, or miR cluster MC‐let‐7a‐1 ~ let‐7d evaluated by the EdU assay (200×). #*P* < .05, ##*P* < .01 vs U87 cells treated with NC inhibitor. The cell experiment was repeated three times. The measurement data were expressed as mean ± standard deviation. Comparisons among multiple groups were tested by one‐way analysis of variance (ANOVA). NC, negative control

The effect of MC‐let‐7a‐1 ~ let‐7d on glioma cell proliferation was subsequently investigated. Compared with the NC mimic, over‐expression of let‐7d, let‐7a‐1, or let‐7f‐1 reduced the cell proliferation of U87 which exhibited a further decrease in the setting of MC‐let‐7a‐1 ~ let‐7d over‐expression (*P* < .01; Figure 3A). Compared with the treatment of NC inhibitor, cell proliferation of U87 was increased after treatment with the inhibitors of let‐7d, let‐7a‐1, or let‐7f‐1, which was further elevated when the U87 cells were treated with the inhibitor of MC‐let‐7a‐1 ~ let‐7d (*P* < .01; Figure 3B). The aforementioned results suggested that up‐regulation of miR cluster MC‐let‐7a‐1 ~ let‐7d suppressed glioma cell proliferation.

### 3.4 Over‐expression of miR cluster MC‐let‐7a‐1 ~ let‐7d promotes cell apoptosis in glioma

**Figure 4:**
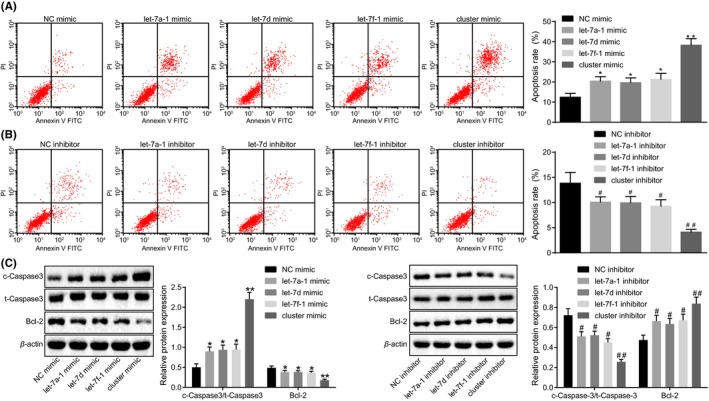
Glioma cell apoptosis is promoted by over‐expression of miR cluster MC‐let‐7a‐1 ~ let‐7d. A, cell apoptosis after over‐expression of let‐7a‐1, let‐7d, let‐7f‐1, or miR cluster MC‐let‐7a‐1 ~ let‐7d evaluated by flow cytometry. B, cell apoptosis after inhibition of let‐7a‐1, let‐7d, let‐7f‐1, or miR cluster MC‐let‐7a‐1 ~ let‐7d evaluated by flow cytometry. C, expression of c‐Caspase‐3, t‐Caspase‐3, and Bcl‐2 after treatment of let‐7a‐1, let‐7d, let‐7f‐1, or miR cluster MC‐let‐7a‐1 ~ let‐7d mimic or inhibitor evaluated by Western blot analysis. *P < .05, **P < .01 vs U87 cells treated with NC mimic. #*P* < .05, ##*P* < .01 vs U87 cells treated with NC inhibitor. The cell experiment was repeated 3 times. The measurement data were expressed as mean ± standard deviation. Comparisons among multiple groups were tested by one‐way analysis of variance (ANOVA). NC, negative control; FITC, fluorescein isothiocyanate; Bcl‐2, B‐cell lymphoma‐2

The effect of MC‐let‐7a‐1 ~ let‐7d on glioma cell apoptosis were evaluated. As evidenced by flow cytometry, the cell apoptosis rate was higher in U87 cells treated with mimics of let‐7d, let‐7a‐1, or let‐7f‐1 than that in U87 cells treated with NC mimic, with the lowest rate of apoptosis detected in the U87 cells treated with miR cluster MC‐let‐7a‐1 ~ let‐7d mimic (*P* < .01; Figure 4A). Consistently, the relative expression of Bcl‐2 was decreased, while the ratio of c‐Caspase‐3/t‐Caspase‐3 was markedly increased following treatment with the mimics of let‐7d, let‐7a‐1, let‐7f‐1, and MC‐let7, respectively, relative to the treatment of NC mimic, which was promoted by miR cluster MC‐let‐7a‐1 ~ let‐7d mimic (*P* < .01; Figure 4C). However, an opposite trend was detected following treatment with the inhibition of let‐7d, let‐7a‐1, let‐7f‐1, or miR cluster MC‐let‐7a‐1 ~ let‐7d (*P* < .01; Figure 4B,C). In conclusion, up‐regulation of miR cluster MC‐let‐7a‐1 ~ let‐7d enhances glioma cell apoptosis.

### 3.5 Over‐expression of miR cluster MC‐let‐7a‐1 ~ let‐7d promotes cell autophagy in glioma

**Figure 5:**
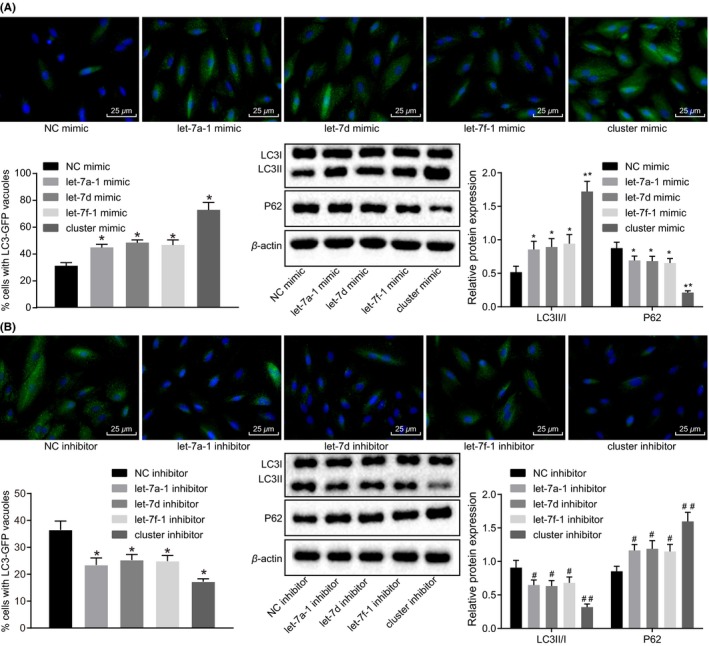
Glioma cell autophagy is increased by over‐expressed miR cluster MC‐let‐7a‐1 ~ let‐7d. A, immunofluorescence staining of U87 cells stably transfected with LC3‐GFP and the protein expression of P62 and LC3Ⅱ/Ⅰ after over‐expression of let‐7a‐1, let‐7d, let‐7f‐1, or miR cluster MC‐let‐7a‐1 ~ let‐7d (400×). B, immunofluorescence staining of U87 cells stably transfected with LC3‐GFP and the protein expression of P62 and LC3Ⅱ/Ⅰ after inhibition of let‐7a‐1, let‐7d, let‐7f‐1, or miR cluster MC‐let‐7a‐1 ~ let‐7d (400×). *P < .05, **P < .01 vs U87 cells treated with NC mimic. #*P* < .05, ##*P* < .01 vs U87 cells treated with NC inhibitor. The cell experiment was repeated three times. The measurement data were expressed as mean ± standard deviation. Comparisons among multiple groups were tested by one‐way analysis of variance (ANOVA). GFP, green fluorescent protein; NC, negative control

The effect of miR cluster MC‐let‐7a‐1 ~ let‐7d on glioma cell autophagy was investigated. As illustrated in Figure 5A, U87 cells treated with mimics of let‐7a‐1, let‐7d, or let‐7f‐1 exhibited enhanced LC3‐positive granules and expression of LC3 II/I protein, and down‐regulated autophagic degradation substrate P62, while the decline was more pronounced following treatment with cluster mimic (*P* < .01). An opposite trend was detected following treatment of inhibition with let‐7d, let‐7a‐1, let‐7f‐1, or miR cluster MC‐let‐7a‐1 ~ let‐7d (*P* < .01; Figure 5B). The aforementioned results suggested that the over‐expression of miR cluster MC‐let‐7a‐1 ~ let‐7d accelerated glioma cell autophagy.

### 3.6 miR cluster MC‐let‐7a‐1 ~ let‐7d represses tumor growth, apoptosis, and autophagy in vivo

**Figure 6:**
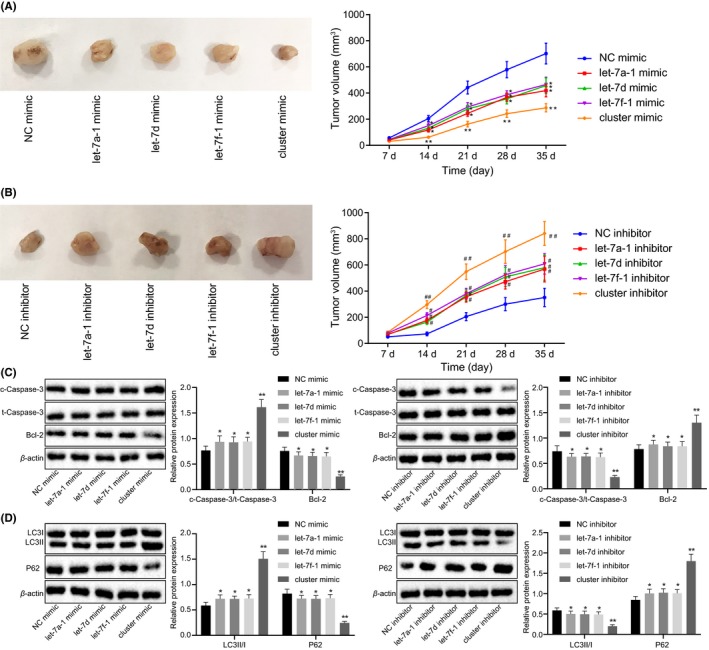
Glioma tumor growth is suppressed by over‐expressed miR cluster MC‐let‐7a‐1 ~ let‐7d in mice. A, the tumors and the growth curves reflecting tumor volume at the indicated time points after over‐expression of let‐7a‐1, let‐7d, let‐7f‐1, or miR cluster MC‐let‐7a‐1 ~ let‐7d in U87 cells. B, the tumors and the growth curves reflecting tumor volume at the indicated time points after inhibition of let‐7a‐1, let‐7d, let‐7f‐1, or miR cluster MC‐let‐7a‐1 ~ let‐7d in U87 cells. C‐D, Western blot analysis of apoptosis and autophagy‐related proteins in tumor tissues following over‐expression of miR cluster MC‐let‐7a‐1 ~ let‐7d. *P < .05, **P < .01 vs mice treated with NC mimic. #*P* < .05, ##*P* < .01 vs mice treated with NC inhibitor. The cell experiment was repeated three times. The measurement data were expressed as mean ± standard deviation. ANOVA of repeated measurement was used in line charts. n = 8. NC, negative control

In order to elucidate the effects of MC‐let‐7a‐1 ~ let‐7d on glioma in vivo*, *in vivo tumorigenicity was performed. Compared with the NC mimic, the tumor growth rate and tumor volume were reduced when treated with mimics of let‐7a‐1, let‐7d, and let‐7f‐1, which was the lowest in the U87 cells treated with cluster mimic (*P* < .01; Figure 6A,B). On the contrary, when the mice were injected with cells transfected with the let‐7 inhibitors, the tumor growth rate and tumor volume were promoted (*P* < .01). We further detected the protein expression of apoptosis and autophagy‐related genes in tumor tissues by Western blot analysis. The results showed that over‐expressed let‐7a‐1, let‐7d, or let‐7f‐1 resulted in elevated protein expression of c‐Caspase‐3/t‐Caspase‐3 and LC3II/I, while decreased Bcl‐2 and P62 protein expression, with the most significant changes observed in the tumor tissues treated with cluster mimic (*P* < .01). A contrasting trend was detected in the aforementioned factors in tumor tissues treated with cluster inhibitor (Figure 6C,D). These results suggested that over‐expression of miR cluster MC‐let‐7a‐1 ~ let‐7d could prevent tumor growth, apoptosis, and autophagy.

### 3.7 STAT3 is targeted by miR cluster MC‐let‐7a‐1 ~ let‐7d directly

**Figure 7:**
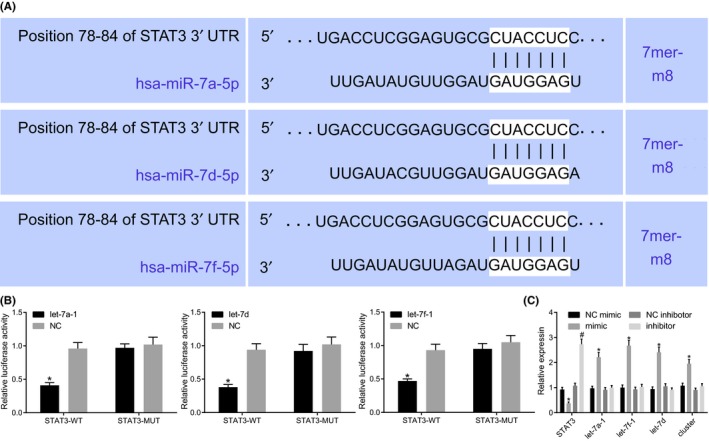
STAT3 is negatively regulated by miR cluster MC‐let‐7a‐1 ~ let‐7d. A, prediction of binding sites between 3’UTR of STAT3 and let‐7a‐1, let‐7d, or let‐7f‐1. B, the binding relationship of STAT3 with let‐7a‐1, let‐7d, and let‐7f‐1 confirmed by the dual‐luciferase reporter gene assay. **P < .05 vs the treatment of NC. C, let‐7a‐1, let‐7d, and let‐7f‐1 affect STAT3 expression ex vivo. *P < .05, **P < .01 vs U87 cells treated with NC mimic. #*P* < .05, ##*P* < .01 vs U87 cells treated with NC inhibitor. The cell experiment was repeated three times. Comparison between two groups was analyzed using unpaired *t* test, and comparisons among multiple groups were tested by one‐way analysis of variance (ANOVA). NC, negative control; STAT3, signal transducers and activators of transcription 3; 3’UTR, 3’untranslated region

Based on the prediction of the direct targeting relationship existing among let‐7d, let‐7a‐1, let‐7f‐1, and the 3’UTR of STAT3, the relationship between STAT3 and MC‐let7 was further investigated and subsequently verified by the dual‐luciferase reporter gene assay (Figure 7A). Following the cotransfection of let‐7 mimics and pGL‐STAT3‐3’UTR‐WT, the luciferase activity of STAT3‐WT was repressed versus the cotransfection with NC. While the cotransfection of let‐7 mimics and pGL‐STAT3‐3’UTR‐MUT exhibited no significant difference between the mimic and the NC (Figure 7B). The results indicated that let‐7d, let‐7a‐1, and let‐7f‐1 could specially bind to STAT3. The functional assays revealed that elevation of let‐7 miRNAs markedly decreased the expression of STAT3. Moreover, silencing of let‐7 miRNAs enhanced STAT3 expression (Figure 7C). Hence, based on our results, we concluded that STAT3 was indeed a target of the miR cluster MC‐let‐7a‐1 ~ let‐7d and could be negatively modulated by the miRNAs.

### 3.8 miR cluster MC‐let‐7a‐1 ~ let‐7d represses cell proliferation while potentiates cell apoptosis and autophagy via down‐regulating STAT3

**Figure 8:**
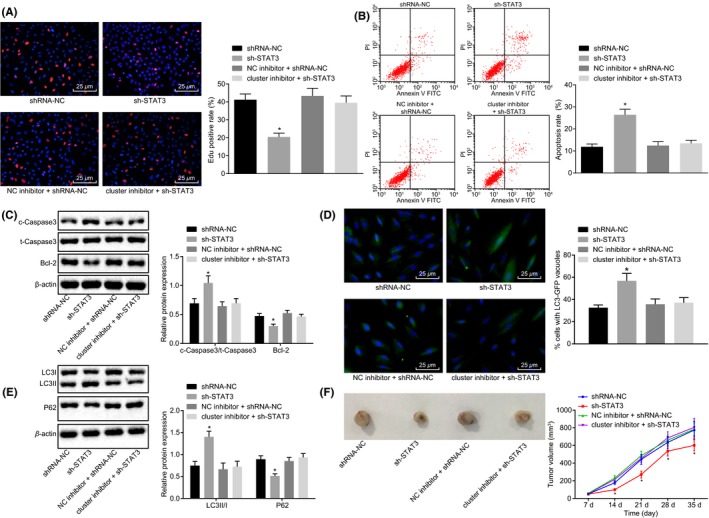
Up‐regulation of miR cluster MC‐let‐7a‐1 ~ let‐7d suppresses cell proliferation but promotes cell autophagy and apoptosis via down‐regulation of STAT3. U87 cells were treated with sh‐STAT3 alone in the presence of cluster inhibitor. A, cell proliferation assessment tested by the EdU assay (400×). B, cell apoptosis measurement after alteration of miR cluster MC‐let‐7a‐1 ~ let‐7d and STAT3 tested by flow cytometry. C, the protein expression of Bcl‐2 and c‐Caspase‐3/t‐Caspase‐3 tested by western blot analysis. D, immunofluorescence staining of U87 cells stably transfected with LC3‐GFP (400×). E, the protein expression of P62 and LC3Ⅱ/Ⅰ tested by Western blot analysis. F, the tumors and the growth curves reflecting tumor volume at the indicated time points (n = 8). *P < .05 vs the treatment of shRNA‐NC. The cell experiment was repeated three times. The measurement data were expressed as mean ± standard deviation. Comparisons among multiple groups were tested by one‐way analysis of variance (ANOVA), and data in panel D were analyzed by ANOVA of repeated measurement. NC, negative control; STAT3, signal transducers and activators of transcription 3; shRNA, short hairpin RNA

The effect of STAT3 on the development of glioma was detected both in vitro and in vivo. The shRNA against STAT3 was then constructed. The expression of STAT3 was reduced by shRNA. Meanwhile, when STAT3 was down‐regulated, cell proliferation was reduced (Figure 8A). In addition, the apoptosis of the glioma cells as well as the ratio of c‐Caspase‐3/t‐Caspase‐3 was increased, whereas the protein expression of Bcl‐2 was diminished as indicated by the flow cytometry results which demonstrated that the expression of the apoptosis‐related proteins after down‐regulation of STAT3 (Figure 8B,C). Moreover, after STAT3 was down‐regulated, LC3 II/I was increased combined with the decrease in autophagic degradation substrate P62, revealing the reduction in cell autophagy induced by silencing of STAT3 (Figure 8D,E). As evidenced by the aforementioned assays, inhibition of MC‐let‐7 remedied the biological effects of STAT3 silencing. The influence of STAT3 on the progression of glioma was assessed in vivo. By silencing of STAT3, the tumor growth rate was reduced with smaller tumor volume (Figure 8F). The above results suggested that STAT3 silencing suppressed glioma cells proliferation and promoted cell apoptosis and autophagy, which was negatively regulated by the miR cluster MC‐let‐7a‐1 ~ let‐7d.

## 4 DISCUSSION

Although great efforts have been made in advancing cancer therapies, the prognosis of glioma remains largely unsatisfactory, particularly in patients with high‐grade glioma.^23^ miRNAs exert a uniquely critical role in a wide variety of cellular processes, including differentiation, development, proliferation, and apoptosis, which has given insight into the link between dysfunction of miRNAs and the pathogenesis of various cancers.^24^ The current study was performed to elucidate the effects of the miR cluster MC‐let‐7a‐1 ~ let‐7d on glioma development. The key findings demonstrated that MC‐let‐7 targets STAT3 and down‐regulates its expression, which ultimately hinders glioma cell proliferation and stimulates the apoptosis and autophagy.

Initially, the present study detected the down‐regulation of let‐7d, let‐7a‐1, and let‐7f‐1 in glioma, which was accompanied by the up‐regulation of STAT3. The let‐7 family is a well‐known suppressor of multiple cancers, which is lost or down‐regulated in a wide variety of carcinomas.^25^ It has been reported that let‐7a is poorly expressed in glioma when compared with that of a normal healthy brain.^26^ Additionally, compared with the adjacent normal tissues, let‐7a expression is found to be reduced in breast cancer tissues.^27^ A previous study presented evidence highlighting the down‐regulation of let‐7f in glioma cells and tissues.^13^ Moreover, Wang et al reported that the expression of MC‐let‐7a‐1 ~ let‐7d was notably lower in HCC cells than that in the immortalized human liver L02 cells,^14^ which was largely consistent with the observations of the current study. Additionally, STAT3 is widely known to be aberrantly up‐regulated in the progression of cancer.^28^ In line with the observations of the current study, accumulating reports have reported the up‐regulation of STAT3 in GBM stem cells and glioma.^29^, ^30^ Furthermore, it has been reported previously that STAT3 activation up‐regulates TRIM8 in order to regulate the stemness in GBM cells.^31^

Another crucial finding of our study illustrated that STAT3 was one of the target genes of let‐7d, let‐7a‐1, and let‐7f‐1. In addition, STAT3 expression was negatively regulated by let‐7d, let‐7a‐1, and let‐7f‐1. As previously reported, miRNAs have the ability to post‐transcriptionally mediate the expression of numerous genes.^32^ Moreover, it has demonstrated that STAT3 is one of the target genes of let‐7a.^15^ A prior study concluded that let‐7a targets and negatively regulates STAT3 in HCC cells.^33^ The targeting relationship between let‐7c and STAT3 in alveolar macrophages has been speculated in previous reports.^34^ Let‐7 exerts an inhibitory effect on the extent of STAT3 phosphorylation and its activation in pancreatic cancer cell lines.^35^ In view of up‐regulation of STAT3 and down‐regulation of let‐7 in glioma, as well as their targeting relationship, let‐7 may influence glioma progression through STAT3.

Cell proliferation was decreased, and cell apoptosis and autophagy were increased in glioma cells after over‐expression of miR cluster MC‐let‐7a‐1 ~ let‐7d. Moreover, STAT3 silencing presented the same biological functions as that of MC‐let‐7 elevation. The over‐expression of let‐7a led to an inhibition of cell proliferation while accelerating glioma cell apoptosis.^26^ Meanwhile, gastric cancer cell autophagy has been shown to be increased in the event of let‐7a up‐regulation.^36^ Besides, let‐7f up‐regulation possesses the capacity to repress cell proliferation, invasion along with migration in glioma.^13^ Additionally, the ectopic expression of let‐7d has been emphasized in relation to its impairment of cell proliferation and boost cell apoptosis in meningioma.^37^ Moreover, the inhibition of STAT3 has been speculated to potentiate glioma cell apoptosis both in animal and cell experiments.^38^ Iwamaru et al asserted that STAT3 silencing promotes metformin‐induced apoptosis and autophagy and then hinders cell viability in esophageal squamous cell carcinoma.^39^ Hence, it was confirmed that MC‐let‐7a‐1 ~ let‐7d constrains cell proliferation while heightening cell autophagy and apoptosis in glioma by down‐regulating STAT3.

This present study demonstrates that STAT3 is conversely mediated and targeted by MC‐let‐7a‐1 ~ let‐7d. Moreover, the over‐expression of MC‐let‐7a‐1 ~ let‐7d was observed to enhance cell apoptosis and autophagy, and impeded cell proliferation in glioma, which was blocked via the down‐regulation of STAT3. Therefore, our results underlined that miR cluster MC‐let‐7a‐1 ~ let‐7d exerts an anti‐oncogenic function on glioma by down‐regulating STAT3. The findings highlight the therapeutic potential of the miR cluster MC‐let‐7a‐1 ~ let‐7d as a possible glioma treatment approach in the future. However, further investigation is still required to confirm the anti‐oncogenic effect of MC‐let‐7a‐1 ~ let‐7d on glioma based on in vivo animal models.

## CONFLICT OF INTEREST

The authors declare no conflict of interest.
