# Supplementary material for: microRNA cluster MC‐let‐7a‐1~let‐7d promotes autophagy and apoptosis of glioma cells by down‐regulating STAT3
Source: CNS Neurosci Ther. 2019 Dec 23;26(3):319–31. doi: 10.1111/cns.13273 (PMC7052808; doi:10.1111/cns.13273)
Supplement: Supplementary file 1 [file CNS-26-319-s001.docx]

**Supplementary Table 1** Clinical characteristics of the 132 patients with glioma enrolled in this study

| Gender | Case |
| --- | --- |
| Male | 74 |
| Female | 58 |
| WHO classification |  |
| grade II | 65 |
| grade III | 45 |
| grade IV | 22 |
| Tumor localization |  |
| frontal lobe | 53 |
| temporal lobe | 40 |
| occipital lobe | 39 |
| Tumor type |  |
| astrocytoma | 68 |
| oligodendroid astrocytoma | 35 |
| glioblastoma | 29 |
